# Transcutaneous Neuromodulation improved inflammation and sympathovagal ratio in patients with primary biliary ssscholangitis and inadequate response to Ursodeoxycholic acid: a pilot study

**DOI:** 10.1186/s12906-020-03036-w

**Published:** 2020-08-01

**Authors:** Hui Yang, Hang Yang, Lixia Wang, Honggang Shi, Bojia Liu, Xue Lin, Qingyong Chang, Jiande D. Z. Chen, Zhijun Duan

**Affiliations:** 1 The Second Department of Gastroenterology, The First Affiliated Hospital of Dalian Medical University, No. 222 Zhongshan Road, Dalian, 116011 Liaoning China; 2 Department of Gastroenterology, West China Hospital, Sichuan University, No. 37, Guo Xue Xiang, Wu Hou District, Chengdu, 610041 China; 3 The Second Department of Neurosurgery, Affiliated Zhongshan Hospital of Dalian University, No. 6 Jiefang Street, Dalian, 116001 Liaoning China; 4 Division of Gastroenterology and Hepatology, Johns Hopkins Center for Neurogastroenterology, Baltimore, MD 21224 USA

**Keywords:** Transcutaneous Neuromodulation, Primary biliary cholangitis, Bile acids, Vagus nerve, Anti-inflammatory

## Abstract

**Background:**

At present, ursodeoxycholic acid (UDCA) is internationally recognized as a therapeutic drug in clinic. However, about 40% Primary Biliary Cholangitis (PBC) patients are poor responders to UDCA. It has been demonstrated that Transcutaneous Neuromodulation (TN) can be involved in gut motility, metabolism of bile acids, immune inflammation, and autonomic nerve. Therefore, this study aimed to explore the effect of TN combined with UDCA on PBC and related mechanisms.

**Methods:**

According to inclusion and exclusion criteria, 10 healthy volunteers and 15 PBC patients were recruited to control group and TN group, respectively. PBC patients were alternately but blindly assigned to group A (TN combined with UDCA) and group B (sham-TN combined with UDCA), and a crossover design was used. The TN treatment was performed via the posterior tibial nerve and acupoint ST36 (Zusanli) 1 h twice/day for 2 weeks. T test and nonparametric test were used to analyze the data.

**Results:**

1. TN combined with UDCA improved the liver function of PBC patients shown by a significant decrease of alkaline phosphatase and gamma-glutamyltransferase (γ-GT) (*P* < 0.05). 2. The treatment also decreased serum IL-6 levels (P < 0.05), but not the level of Tumor Necrosis Factor-α, IL-1β or IL-10. 3. TN combined with UDCA regulated autonomic function, enhanced vagal activity, and decreased the sympathovagal ratio assessed by the spectral analysis of heart rate variability (*P* < 0.05). 4. There was no change in 13 bile acids in serum or stool after TN or sham-TN.

**Conclusions:**

TN cssombined with UDCA can significantly improve the liver function of PBC patients. It is possibly via the cholinergic anti-inflammatory pathway. TN might be a new non-drug therapy for PBC. Further studies are required.

**Trial registration:**

The study protocol was registered in Chinese Clinical Trial Registry (number ChiCTR1800014633) on 25 January 2018.

## Background

Primary Biliary Cholangitis (PBC) is a progressive autoimmune cholestatic liver disease. Main pathological features are chronic inflammation, the destruction of small intrahepatic bile ducts, and progressive cholestasis toward cirrhosis and liver failure [1]. Patients with PBC may even die of its complications [2]. In recent years, the incidence of PBC has increased substantially [3]. However, there are few drugs and treatment options for PBC. Ursodesoxycholic acid (UDCA) is the only standard first-line therapy for PBC, but it is expensive and about 40% of patients have an inadequate response to it [3, 4]. Therefore, it is necessary to search for new and effective treatments to delay or even reverse the progress of PBC in the early stage.

Transcutaneous Neuromodulation (TN), using Needleless Transcutaneous Electrical Acustimulation, has recently received increasing attention. For its safety and reliability, TN is widely used in diseases related to immune and inflammatory injury, such as rheumatoid arthritis (implanted vagus nerve stimulation) [5] and inflammatory bowel disease (implantable vagus nerve stimulation) [6], as well as functional gastrointestinal diseases [7], such as functional dyspepsia (stimulation of Zusanli and Neiguan points) [8] and functional constipation (stimulation of Zusanli and posterior tibial nerve) [9]. It was suggested to improve major postoperative symptoms by enhancing vagal and suppressing sympathetic activities to promote post-operative recovery [10]. TN was also reported to treat liver diseases, such as acute liver injury (stimulation of Zusanli points), hepatitis (stimulation of Zusanli points), and non-alcoholic fatty liver (stimulation of Zusanli points) [11]. TN as a non-invasive therapy is more acceptable to patients [11]. In our previous study, TN could improve the excitability of the vagus nerve and inhibit the synthesis and release of inflammatory factors. TN could also regulate the level of gastrointestinal hormone and the metabolism of serum bile acids (BAs) [12]. Moreover, stimulation at both Zusanli and posterior tibial nerve can maximize the advantages of TN [9, 12, 13]. Other studies have found that stimulation at Zusanli can inhibit inflammation and regulate the immune function of patients [11, 14].

Patients with PBC have abnormal bile acid metabolism, which is characterized as increased BAs contents, changes in bile acid composition, and the abnormal expression of bile acid transporters [15–18]. The toxic accumulation of bile acid may be related to the progression of PBC. In addition, the level of helper T cells in PBC patients is increased, secreting interleukin-6 (IL-6), interleukin-10 (IL-10), and tumor necrosis factor-α (TNF-α). Through the secretion of bile, the inflammatory factors secreted by liver can enter intestine, and aggravate the damage of intestinal lining and disrupt microbiota. Then, liver is re-damaged by the gut-liver circulation [19].

As TN has been reported to play a role in regulation of gut motility, gut secretion, metabolism of bile acids, immune inflammation, and autonomic nerve as mentioned above. We hypothesized that TN may influence PBC through the anti-inflammatory effect of the vagus nerve and the regulation of bile acid according to the characteristics of PBC.

The aim of this study was to investigate the improvement of PBC with TN combined with UDCA, and to explore whether the anti-inflammatory effect of vagus nerve and the regulation of bile acid were involved in the mechanism of TN in the improvement of PBC.

## Methods

### Study participants

Fifteen PBC patients and 10 healthy volunteers (HV) were recruited from the Second Department of Gastroenterology, the First Affiliated Hospital of Dalian Medical University from January 2018 to January 2019. Data was obtained for 10 PBC and 10 HV. The study protocol was approved by the hospital ethics committee (number PJ-KY-2017-113(X)) and registered in Chinese Clinical Trial Registry (number ChiCTR1800014633). All the participants in this study signed the informed consent form and were free to quit this study at any time for any reason.

Inclusion criteria for patients were as follow: According to the European Association for the Study of the Liver Clinical Practice Guidelines: Diagnosis and Management of PBC Patients [20]. Meeting two of the following: (1) a. biochemical evidence of cholestasis, levels of alkaline phosphatase (ALP) and γ-glutamyltransferase (γ-GT) were increased; b. serologic reactivity to anti-mitochondrial antibodies (AMA) and/or AMA-M2; c. Liver histology evidence: chronic non-suppurative, granulomatous, and lymphocytic small bile duct cholangitis. (2) The level of serum total bilirubin is within the normal range. Exclusion criteria were as follow: (1) symptoms of pruritus; (2) liver elastography as stage F4 or cirrhosis; (3) other liver diseases or other immune diseases; (4) being in acute phase; (5) the level of liver function was in the normal range, BMI > 35 kg/cm^2^; (6) having an implanted pacemaker; (7) being allergic to skin preparation or electrodes; (8) pregnancy or intention to become pregnant during the trial; (9) refusal of blood and fecal withdrawing; (10) being familiar with acupoints or meridian.

### Study design

**Fig. 1:**
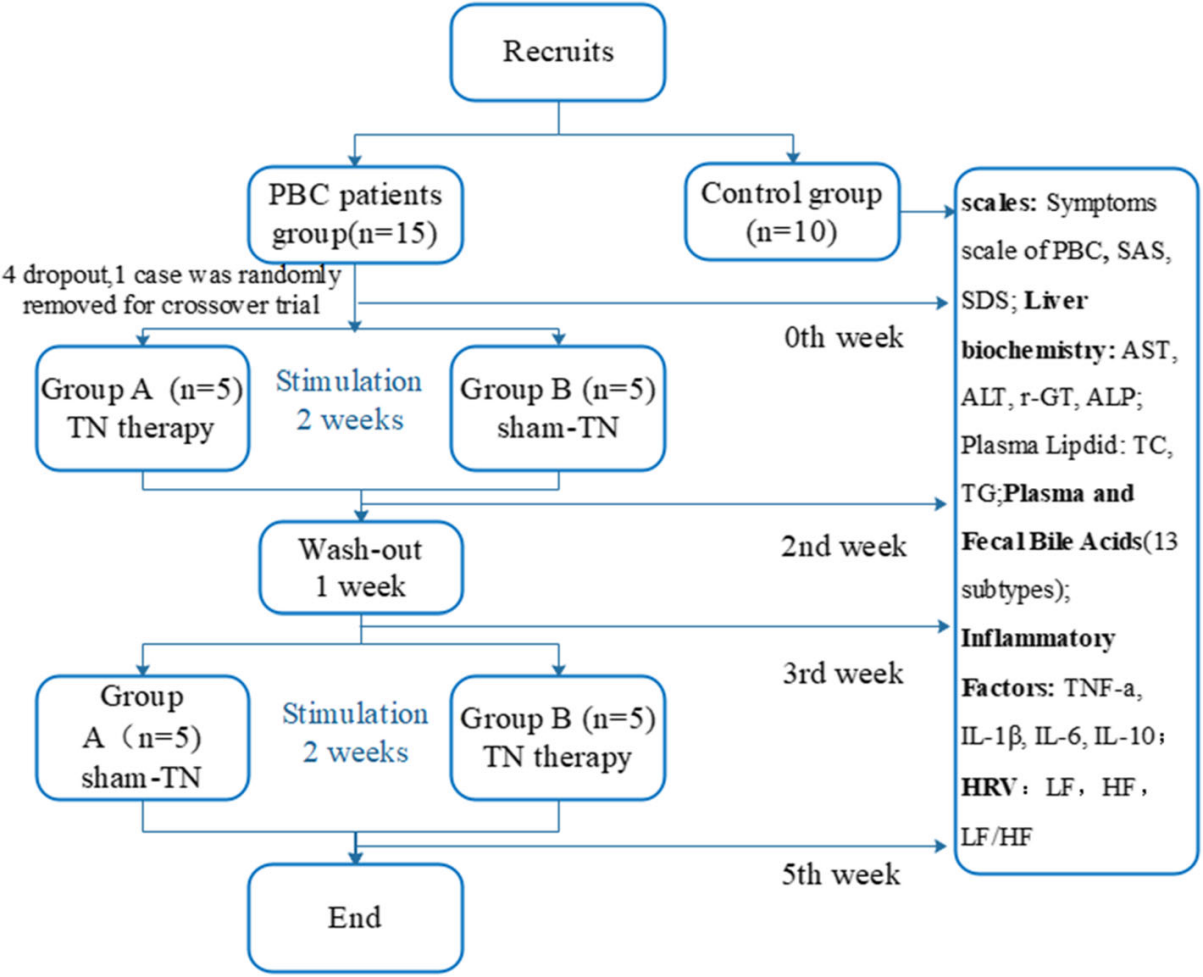
Experimental protocols

All subjects were enrolled according to inclusion and exclusion criteria. Healthy control group: blood samples were taken and electrocardiogram was recorded for assessing Heart Rate Variability (HRV). PBC group: baseline conditions were recorded, including HRV, blood samples, and fecal specimens. PBC is a chronic disease. In our study patients had an inadequate response to UDCA and the status of their disease had not changed much in a short time. Therefore, the experiment used a crossover design. PBC patients were blinded about treatment regimens. They did not know which kind of plan they received, and did not know the existence of sham TN, as well as sham TN treatment was useless. All the patients were alternately but blindly divided into two groups (Fig. 1): one group was allowed to cross over to sham-TN after receiving 2-week TN therapy followed by 1-week washout; the other group was allowed to cross over to TN therapy after receiving 2-week sham-TN followed by 1-week washout. For methods, we made the treatment plan for the first patient starting with TN treatment combined with UDCA, and the second patient starting with sham-TN treatment combined with UDCA, which is alternate in sequence. Patients were required for three office visits (2nd, 3rd, and 5th week) during the trial. In each visit, blood and fecal samples were taken, and HRV were recorded. At the end of the trial, the data of two groups were regrouped. The data of patients receiving TN therapy were classified into TN group, and the data of patients receiving sham-TN were classified into sham-TN group. During the study period, patients continued to take UDCA at the same dose as before the study. (Fig. 1).

#### TN treatment

**Fig. 2:**
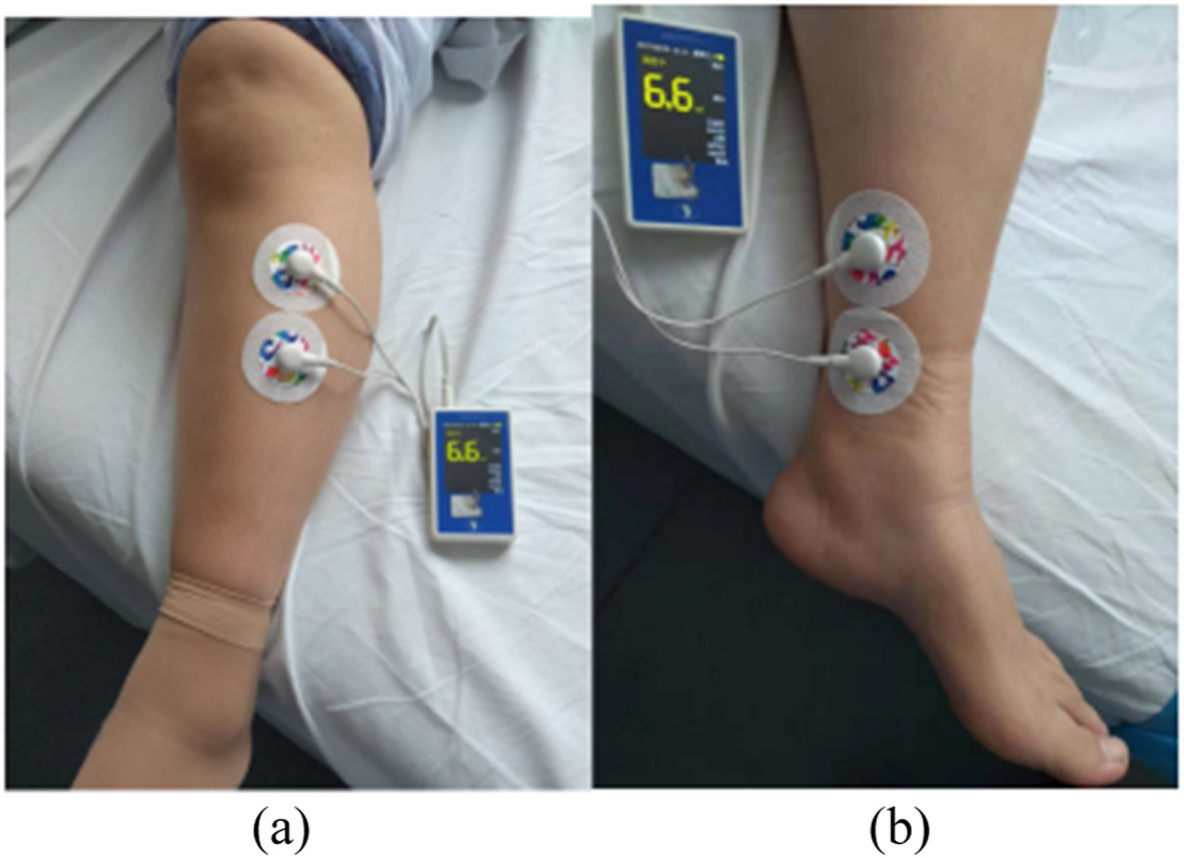
Method of TN. **a** TN acupoints: ST36-one electrode was placed at ST36 and the other at 4 cm below ST36 along the same meridian; the posterior tibial nerve-one electrode was placed at about two fingers’ breadth up to the malleolus medialis and posterior to the tibia and the other at 4 cm above the first electrode. **b** Sham-TN acupoints: for ST36 was located at about 10 cm away from ST36 and to the lateral side of ST36 not on any meridian and for the posterior tibial nerve was located at about 10 cm away from and to the lateral side of the posterior tibial nerve not on any meridian

A neuromodulator (SNMFDC01, Ningbo Maida Medical Device Inc., Ningbo, China), a watch-size microstimulator, was used for TN treatment and sham-TN. TN treatment was delivered via electrodes placed at the posterior tibial nerve and acupoint ST36 (Zusanli) (Fig. 2). For ST36, one electrode was placed at ST36 and the other at 4 cm below ST36 along the same meridian; for the posterior tibial nerve, one electrode was placed at about two fingers’ breadth up to the malleolus medialis and posterior to tibia and the other at 4 cm above the first electrode. The sham-point for ST36 was located at about 10 cm away from ST36 and to the lateral side of ST36 not on any meridian and the sham-point for the posterior tibial nerve was located at about 10 cm away from and to the lateral side of the posterior tibial nerve not on any meridian. TN treatment was given for 1 h, twice daily (9–10 a.m. and 4–5 p.m.). Following parameters were used for TN and sham-TN including train on-time of 10 s and of-time of 90 s, pulse width of 0.5 ms, pulse frequency of 25 Hz, and amplitude of 2–10 mA (at the maximum level tolerated by the subject). (Fig. 2).

#### Blood draw and assay

Blood samples were taken in fasting state (7–8 am) from healthy volunteers and PBC patients (weeks 0,2,3,5, Fig. 1). (1) Liver function: ALT, AST, ALP, and γ-GT were assessed by using an automatic biochemical analyzer (Hitachi 7600_020 biochemical automatic analyzer). (2) Serum lipid: using automatic biochemical analyzer (Hitachi 7600_020 biochemical automatic analyzer) to determine total cholesterol (TC) and triglyceride (TG). (3) Serum BAs: five standard products were accurately weighed and formulated into standard stock solutions with a concentration of 1 mg/ml, which were sequentially diluted with matrix to a mass concentration of 0.2, 1, 5, 10, 50, 100, 500,1000 ng/ml total eight series of standard solutions [12]. Cryopreserved blood samples were lyophilized and reconstituted with a methanol solution. LC-MS/MS was used to detect 13 subtypes of BAs: tauroursodeoxycholic acid (TUDCA), taurocholic acid (TCA), taurochenodeoxycholic acid (TCDCA)), taurodeoxycholic acid (TDCA), chenodeoxycholic acid (CDCA), UDCA, cholic acid (CA), lithocholic acid (LCA), taurolithic acid (TLCA), glyco-eodeoxycholic acid acid (GCDCA), glycocholic acid (GCA), glycodeoxycholic acid (GDCA), deoxycholic acid (DCA). (4) Inflammatory Factors: blood samples were taken from a refrigerator at − 80 °C and thawed at 4 °C. TNF-a, IL-6, interleukin-1β (IL-1β), and IL-10 were detected by Immulite 1000 automatic immunoassay analyzer (Siemens) and its supporting reagents. (5) Fecal samples were taken in fasting state (7–8 am) from healthy volunteers and PBC patients (0th, 2nd, 3rd, and 5th week) and saved in a − 80 °C refrigerator. Detection methods and BAs types were the same as serum BAs.

#### Assessment of autonomic function

Autonomic function was assessed by the spectral analysis of HRV derived from ECG, described as follows: three locations in chest/abdomen where ECG electrodes were placed (apical, the second intercostal of sternal right edge, and the intersection of right rib arch and clavicle midline) and marked. Abdominal skin was cleaned and a medical ultrasound gel (Dafuxingyejingsheng science and technology limited company, Beijing, China) was applied to reduce skin impedance [21]. Electrodes were placed over the treated skin, and patients were asked to lie down and stay quiet for 30 min. During the examination, patients could not talk, move, or sleep, to avoid interference such as motion artifacts. ECG was recorded using an amplifier (ECG-201, Ningbo Maida Medical Device Inc., Ningbo, China) with a recording frequency of 100 Hz. HRV signal was obtained by identifying R peak, and RR interval from ECG was calculated by using a custom-made software [9]. HRV signal was subjected to power spectrum analysis and the power in different bands was calculated. The power in low-frequency (LF) band (0.04–0.15 Hz) reflects mainly sympathetic activity, whereas the power in high-frequency (HF) band (0.15–0.40 Hz) represents purely parasympathetic or vagal activity. And the power in LF/HF represents the balance of sympathetic-parasympathetic [21, 22].

### Statistical analysis

Statistical analysis was conducted by using SPSS 20.0 Software. Data with a normal distribution are presented as mean ± SD. Data between groups was analyzed by independent sample t test, and Data within groups was tested by paired sample t test (AST, ALT, ALP, TC, TG, LF, and HF). Data that did not have a normal distribution were presented as median (quartile range) and analyzed by nonparametric test (γ-GT, TNF-α, IL-6, and LF/HF). Statistical significance was assigned for *p* < 0.05.

## Results

Fifteen PBC patients (13 females, 2 males) were enrolled in the study. Four patients dropped out during the study due to poor compliance (incomplete collection of samples). None of patients reported any adverse reactions such as changes in stool frequency, palpitations, or sweating. No patients had allergic reactions to stimulation electrodes. Eleven patients underwent the trial without any uncomfortable complaints. Because the crossover experiment required an even sample size, 1 case was randomly removed. A total of 10 patients (8 females and 2 males) and 10 healthy volunteers (7 females and 3 males) were included in statistical analysis. As shown in Table 1, there were no differences noted between patients and controls in age, weight, height, or body mass index (BMI) (*p* > 0.05).

**Table 1:** Characteristic of subjects (mean ± SD)

	male/female	age	height (cm)	weight (kg)	BMI
PBC	2/8	52.10 ± 11.10	164.30 ± 6.88	66.15 ± 10.15	24.41 ± 2.61
Control	3/7	47.20 ± 8.60	166.90 ± 8.63	66.10 ± 7.92	23.73 ± 1.87

P1 is PBC patients (pretreatment) compared with controls, *p < 0.05.

### Liver biochemistry

As shown in Table 2, compared with controls, the level of serum AST, ALT, ALP, and γ-GT were significantly higher in PBC patients (0th week) than in HV (p < 0.05). The level of serum ALP and γ-GT in PBC patients were decreased significantly after TN combined with UDCA (*P* < 0.05), but there was no significant difference after sham-TN combined with UDCA (*P* > 0.05). However, after either TN or sham-TN treatment, there was no significant change in serum AST and ALT (P > 0.05).

**Table 2:** Assessment of Liver Biochemistry and Lipids

	Control	PBC (0th week)	TN	sham-TN		
			before	after	before	after
AST (U/L, mean ± SD)	**23.30 ± 4.57**	**37.70 ± 12.90^**	37.10 ± 14.71	36.50 ± 16.71	34.70 ± 14.97	36.80 ± 19.62
ALT (U/L, mean ± SD)	**16.30 ± 6.41**	**47.30 ± 23.16^**	46.40 ± 27.35	38.30 ± 25.17	38.80 ± 24.49	42.70 ± 35.24
ALP (U/L, mean ± SD)	**62.00 ± 15.36**	**204.40 ± 94.51^**	**191.40 ± 70.42**	148.50 ± 49.66*	178.40 ± 99.61	165.30 ± 65.75
γ-GT (U/L, mean ± SD, Median50(M25 ~ M75))	**22.20 ± 8.53**	**287.60 ± 176.38^**	**217.00 (126.75 ~ 418.50)**	138.50 (102.50 ~ 302.25)*	165.00 (111.50 ~ 348.25)	151.50 (91.75 ~ 314.75)
TC (mmol/l, mean ± SD)	**4.58 ± 0.53**	**5.87 ± 1.59^**	6.01 ± 1.27	5.94 ± 1.68	5.78 ± 1.83	6.05 ± 1.25
TG (mmol/l, mean ± SD)	1.06 ± 0.50	1.32 ± 0.66	1.43 ± 0.69	1.42 ± 0.37	1.28 ± 0.4	1.28 ± 0.45

n = 10; p1 is PBC patients (pretreatment) compared with controls, ^p < 0.05; p2 is the comparison of before and after TN/sham-TN, *p < 0.05. TC = total cholesterol, TG = Tryglcerides**.**

### Lipid changes

As shown in Table 2, compared with control group, total cholesterol (TC) in the PBC patients (pretreatment) was higher (*P* < 0.05), while the level of triglycerides (TG) was not different with HV (*P* > 0.05). Neither TN nor sham-TN treatment resulted in a significant change (P > 0.05) in TC or TG.

### Modulation of bile acid metabolism

#### Plasma bile acids

**Fig. 3:**
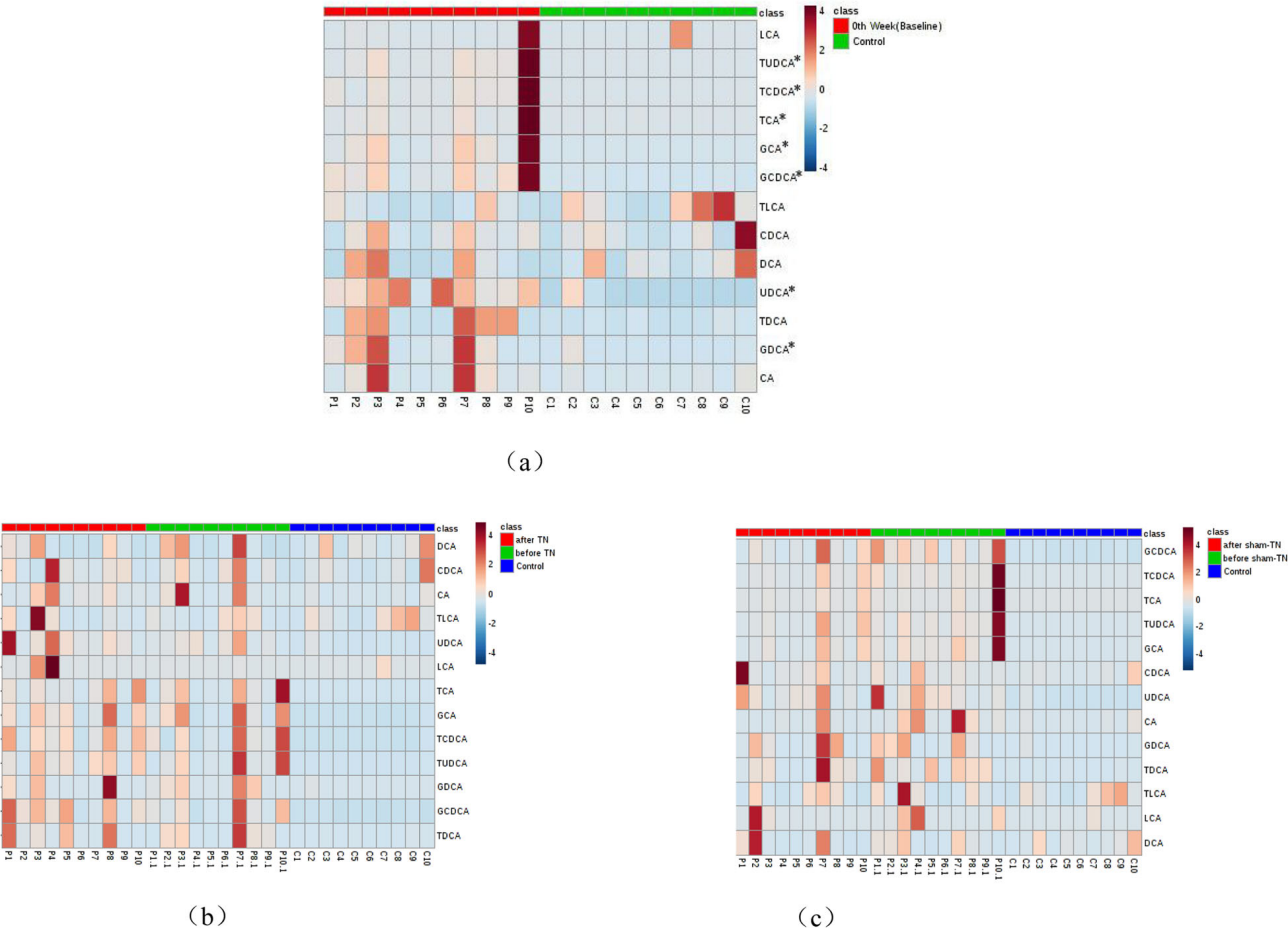
Modulation of Plasma Bile Acid Metabolism. **a** Comparison of plasma BAs in the PBC (pretreatment) group to the control group. After statistical analysis, TCDCA, TUDCA, UDCA, GCA, GDCA, GCDCA and TCA were significantly higher in the PBC patients (pretreatment) than the controls (*P* < 0.05), and the color bars for the BAs in PBC group were browner indicating a higher level. (− 4 < = log_2_ (x) < = 4. It is obtained from the heat map website: https://www.metaboanalyst.ca/). **b** Comparison of plasma BAs in the PBC group before (P1–10) and after TN combined with UDCA (P1.1–10.1) compared with the control group (C1–10). After TN combined with UDCA, bile acids had no significant change to pretreatment. The color bars look similar. Visually, this indicated no obvious changes in the metabolism of serum bile acids after TN combined with UDCA. **c** Comparison of plasma BAs in the PBC group before (P1–10) and after sham-TN with UDCA (P1.1–10.1) with control group (C1–10). After Sham TN combined with UDCA, bile acids had no significant change to pretreatment. The color bars look similar. Visually, this indicated no obvious changes in the metabolism of serum bile acids after sham TN combined with UDCA

As shown in Fig. 3, TCDCA, TUDCA, UDCA, GCA, GDCA, GCDCA, and TCA were significantly higher in PBC patients (pretreatment) than those in controls (P < 0.05). After TN combined with UDCA, 13 subtypes of bile acids had no significant change (P > 0.05). After sham-TN combined with UDCA, the level of TUDCA and GCA showed a downward trend (*P* = 0.093, *P* = 0.053). There was no significant change in the remaining 11 subtypes of bile acids (*P* > 0.05). These results indicate there was no obvious changes in the metabolism of serum bile acids after TN combined with UDCA.

#### Fecal bile acid

**Fig. 4:**
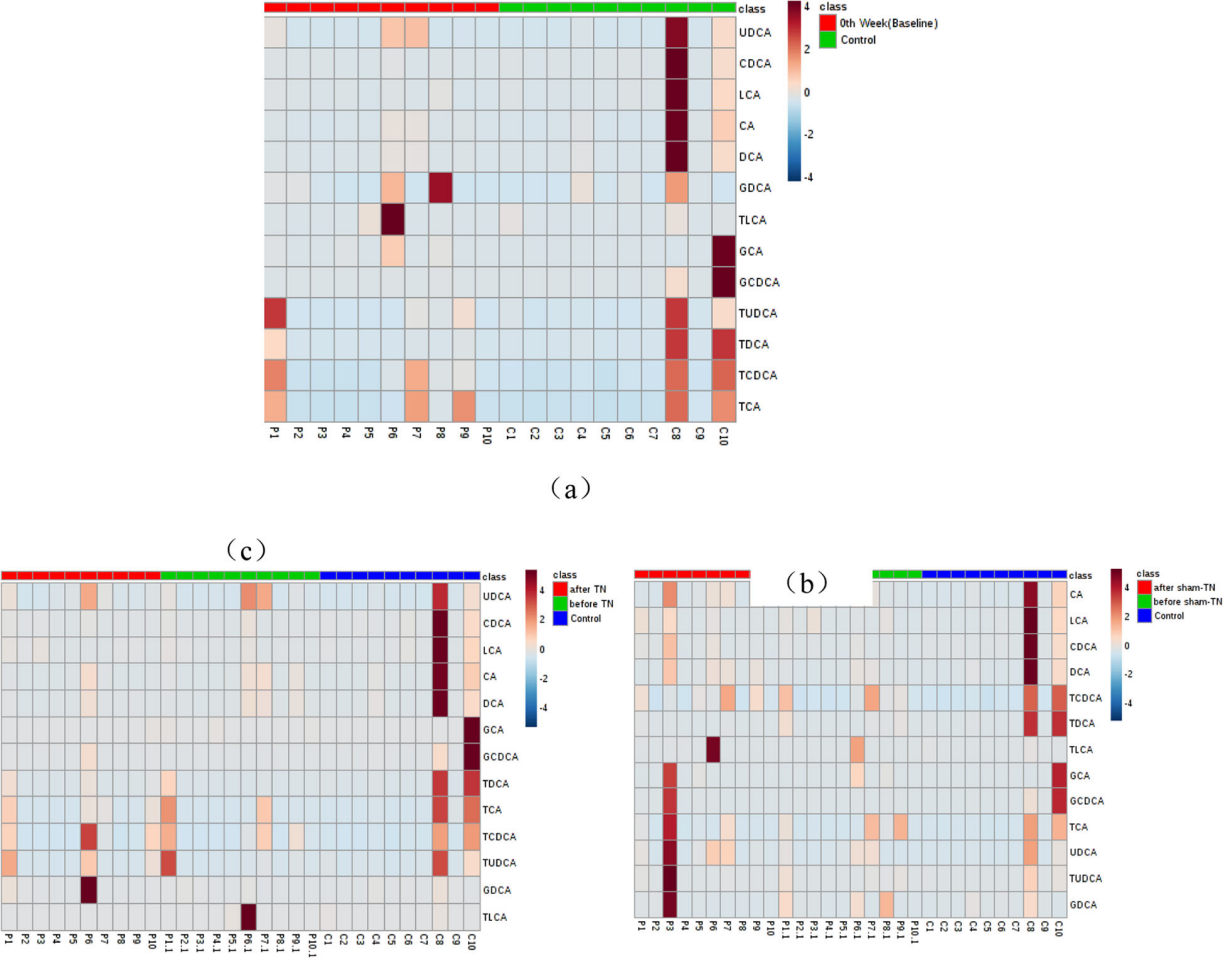
Modulation of Fecal Bile Acid Metabolism. **a** Comparison of fecal BAs in the PBC (pretreatment P1–10) to the control group (C1–10). **b** Comparison of fecal BAs in the PBC group before (P1–10) and after TN (P1.1–10.1) and the control group (C1–10). After TN combined with UDCA, bile acids had no significant change to pretreatment. The color bars look similar. **b** Comparison of fecal BAs in the PBC group before (P1–10) and after sham-TN (P1.1–10.1) and the control group (C1–10). After Sham TN combined with UDCA, bile acids had no significant change to pretreatment. The color bars look similar. After statistical analysis, there were no significant changes in fecal BAs. The color bars in three heatmap were also look similar. It indicated no obvious changes in the metabolism of fecal bile acids after TN combined with UDCA visually

As shown in Fig. 4, compared with controls, the level of CDCA in PBC patients (pretreatment) showed a non-significant decrease (*P* = 0.199) and GCA showed a non-significant increase (*P* = 0.103). There was no significant change in the remaining 11 subtypes of bile acids. After TN combined with UDCA, 13 subtypes of bile acids showed no significant changes (*P* > 0.05). After sham-TN combined with UDCA, the level of CA and DCA showed an increasing trend (*P* = 0.074, *P* = 0.059), and the remaining 11 subtypes of bile acids showed no significant changes (P > 0.05). These results indicated there was no obvious change in the metabolism of fecal bile acids after TN or sham-TN combined with UDCA.

### Alteration in plasma inflammatory factor

**Fig. 5:**
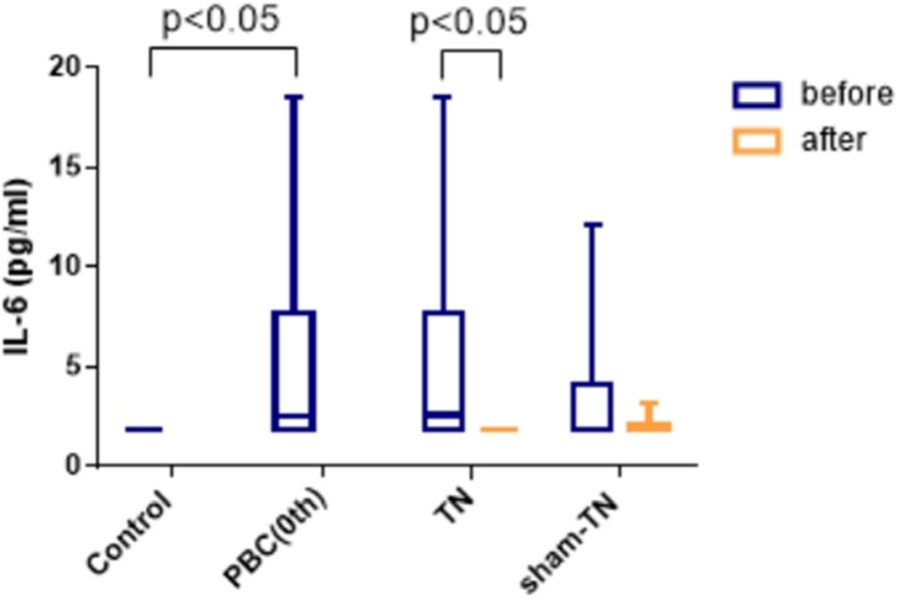
Alteration in IL-6(plasma inflammatory factor). IL-6 in the control group, PBC group (pretreatment), before and after TN and before and after sham-TN). (*n* = 10 for each group)

In this study, four types of inflammatory factors, TNF-α, IL-1β, IL-6, and IL-10 were determined. Plasma levels of IL-1β and IL-10 were less than 5.00 pg/ml in healthy controls and PBC patients. After TN or sham-TN combined with UDCA, plasma levels of IL-1β and IL-10 showed no changes. Compared with controls, plasma levels of TNF-α (40.36 ± 43.85 pg/ml) and IL-6 (7.43 ± 6.58 pg/ml) were significantly increased in PBC patients (pretreatment) (*P* < 0.05). Plasma level of TNF-α in PBC patients (3.90(3.90 ~ 42.65) pg/ml) after TN combined with UDCA (less than 4 pg/ml) showed a trend of decrease (*P* = 0.068), which was not seen after sham-TN combined with UDCA (3.90(3.90 ~ 14.70)pg/ml) (*P* > 0.05). The level of IL-6 was decreased significantly after TN combined with UDCA (*P* < 0.05) but not significantly changed after sham-TN combined with UDCA (P > 0.05) (Fig. 5).

### Autonomic mechanisms of TN

**Fig. 6:**
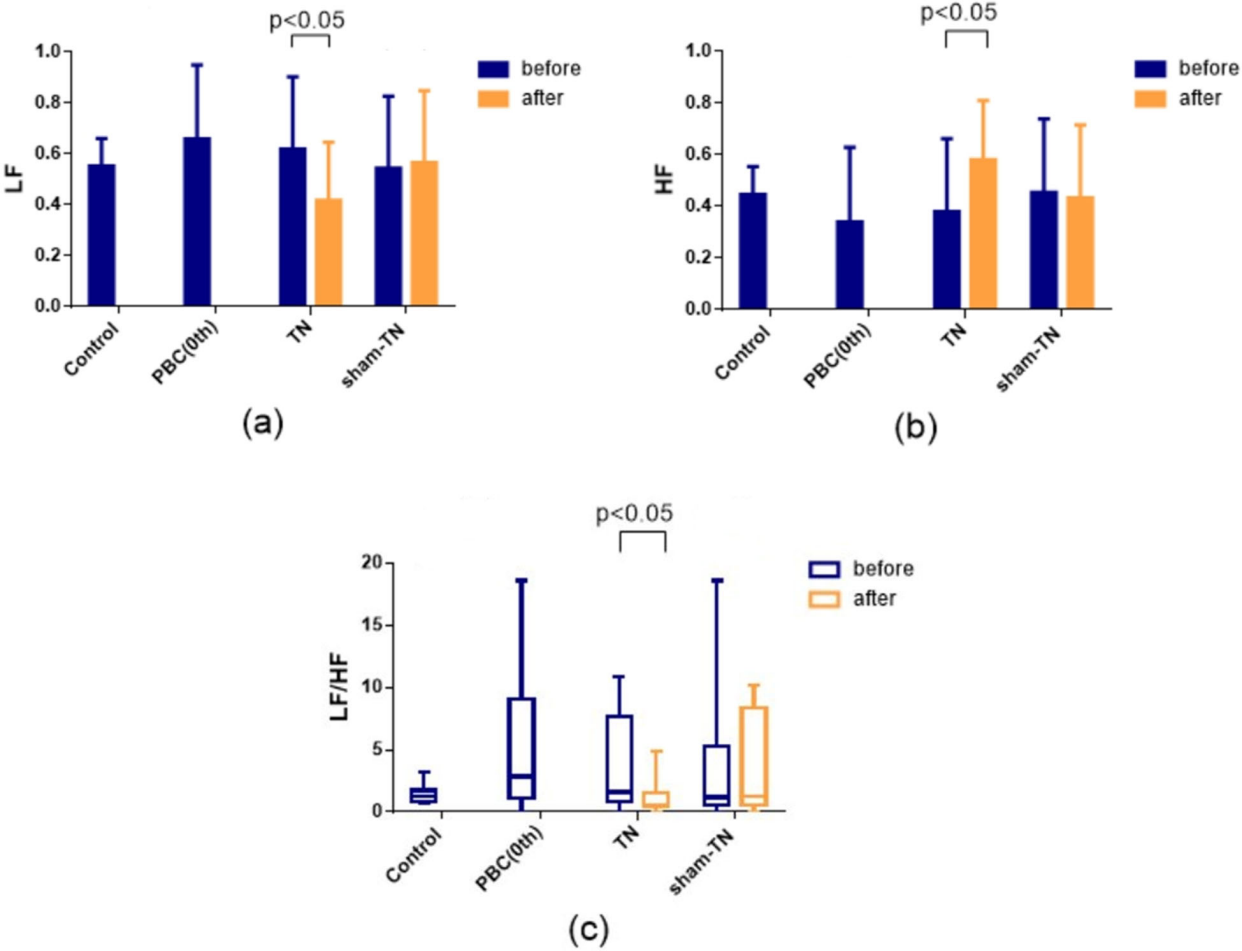
Analysis of Heart Rate Variability (HRV). **a** Low frequency component (LF) in the control group, PBC group (pretreatment), before and after TN, and before and after sham-TN); **b** High frequency component (HF) in the control group, PBC group (pretreatment), before and after TN, and before and after sham-TN); **c** LF/HF ratio in the control group, PBC group (pretreatment), before and after TN, and before and after sham-TN). (n = 10)

As shown in Fig. 6, LF (reflecting sympathetic activity), HF (reflecting vagal activity) and LF/HF in the PBC patients (0th week) showed no significant difference to HV (P > 0.05). After TN/UDCA but not after sham-TN/UDCA (P > 0.05), LF was significantly decreased, HF was significantly increased (P < 0.05) and LF/HF were significantly decreased (P < 0.05). These results indicated that TN therapy could enhance vagal excitability or decrease sympathetic activity.

## Discussion

The potential therapeutic effect of TN was investigated in this study in PBC patients. TN combined with UDCA significantly reduced ALP and γ-GT in PBC patients. In addition, TN combined with UDCA significantly decreased sympathetic activity and sympathovagal ratio, and increased vagal activity without significant improvement in circulating and stool bile acids. These data suggested that TN combined with UDCA might improve the inflammatory process of PBC patients possibly via the cholinergic anti-inflammatory pathway [23].

With the application of UDCA in the treatment of PBC patients, the prognostic efficacy of ALP and other biochemical markers, which are the secondary indicators to replace primary endpoints of death and liver transplantation, continues to be evaluated [24–26]. In recent years, large-scale cohort studies of PBC in two groups have shown that whether used to assess PBC under discrete threshold [27] or as a continuous variable [28], lower serum ALP levels combined with other biochemical markers are associated with higher survival rate (no liver transplantation). In the study, we found that TN combined with UDCA significantly reduced serum ALP and γ-GT levels in PBC patients (*P* < 0.05), but not sham-TN combined with UDCA. This suggests that TN combined with UDCA is effective in the treatment of PBC patients with poor UDCA responses.

Toxic accumulation of bile acids in hepatocytes of PBC patients can induce oxidative stress and further apoptosis, leading to the damage of liver parenchyma [29, 30]. Therefore, the reduction of bile acids is a pivotal therapeutic target for PBC [31]. A comprehensive analysis of bile acid profiles of PBC patients becomes more and more important. TCDCA and GCA are positively correlated with changes of PBC stages [16]. This is consistent with results of significantly higher glycine-taurine conjugated bile acids of PBC patients than those of healthy subjects. Taurine and glycine-bound bile acids are hydrophobic molecules, showing similar cytotoxicity [32–35]. But compared with glycine-bound bile acid, taurine-bound bile acid can also activate cell survival and anti-apoptotic pathways, which can prevent its inherent toxicity [36, 37]. However, in this study, TN treatment did not significantly improve the bile acid profile of PBC, which may be related to the long-term oral administration of UDCA and the small number of participants. Therefore, the regulatory effect of TN on bile acid metabolism of PBC needs to be further explored, such as study on PBC patients who do not take UDCA.

Although the multifaceted and complex pathogenesis of PBC is not fully clear yet, autoimmune mechanism is internationally recognized as an essential factor [38]. The immune response of PBC is highly specific to bile duct epithelium [39]. Persistent congenital and adaptive immune response to intralobular bile duct and chronic inflammation, and further lead to bile duct destruction and cholestasis. In PBC patients, it is generally believed that the increase of T helper type 1 cell (Th1) and T helper type 2 cell (Th2) ratio is one of the most important factors in the pathogenesis of the disease [40, 41]. Some studies have shown that a variety of cytokines related to Th1, Th2, and T helper type 17 cell (Th17) are also involved [42]. Th1 mainly produces IL-2, IFN-γ, TNF-α, and Th2 mainly produces IL-4, IL-6, IL-10 and so on. Stimulating vagus nerve can inhibit inflammatory factors such as TNF, IL-1, and IL-6 released by macrophages [23, 43], which may represent a novel approach. In other research, they showed TN could excite vagus nerve and decrease TNF-α in postoperative patients [10]. Recent studies have found that IL-6 plays a promotive role in the occurrence and development of various autoimmune diseases [44, 45]. A variety of immune responses are suggested to be regulated by CD4+ T cells. Firstly, IL-6 promotes the differentiation of Th2 and upregulates the expression of its related inflammatory factors, thereby regulating the ratio of Th1/Th2 [46]. Secondly, IL-6 can promote the differentiation and maturation of Th17 cells together with other cytokines such as transforming growth factor-beta (TGF-β), IL-23, and IL-1β [47]. Therefore, four inflammatory factors, TNF-α, IL-1β, IL-6 and IL-10, were measured in PBC patients before and after treatment. After TN combined with UDCA, the level of IL-6 in serum was significantly lower than that before treatment, but there was no significant difference before and after treatment with sham-TN combined with UDCA. The results suggest that IL-6 may be involved in the occurrence and development of PBC. And TN combined with UDCA could improve PBC via decreasing IL-6.

HRV is a simple and non-invasive examination, which can evaluate the activity of autonomic nerve, the energy-saving ability of the body, and the influence of interventions on the function of autonomic nerve in patients [48, 49]. Previous studies have shown nearly 70% of PBC patients with autonomic nervous dysfunction [50]. Some studies suggest that 24 h-HRV analysis is more sensitive in detecting parasympathetic and sympathetic nerve, and autonomic nervous dysfunction is associated with the duration and severity of PBC [51]. Previous experiments in our group demonstrated that TN could increase vagal activity and restore sympathetic vagal balance [9]. In this study, we found that TN combined with UDCA therapy changed the HRV of PBC patients, which once again proved that TN could excite the vagal pathway. Vagus nerve plays a key role in anti-inflammatory cholinergic pathway. Stimulating the vagus nerve can release acetylcholine [52, 53]. The release of acetylcholine can bind to the alpha-7-nicotinic acetylcholine receptor (alpha-7nAChR) of macrophages (such as Kupffer cells of the liver), thus inhibiting the release of inflammatory factors such as TNF-α, IL-1, IL-6 by macrophages [23, 54, 55]. Therefore, TN can activate the cholinergic anti-inflammatory pathway by increasing the activity of vagus nerve, thereby inhibiting the release of inflammatory factors, and ultimately improving the inflammatory response of PBC patients. On one hand, it can provide a new and feasible non-invasive treatment as a supplement to the clinical treatment of PBC. As patients with PBC were in early stage with response to the UDCA, TN combined with UDCA showed significant effect on LF/HF ratio. So, it is reasonable to test PBC patients with poor response to UDCA in a future clinical trial. On the other hand, this is the first suggestion that the cholinergic anti-inflammatory pathway may have a role in treating PBC, and provides a new idea on the mechanism of PBC. In this study, TN was used as a combination therapy with UDCA. Whether TN can be used alone in the treatment of PBC patients need further verification. And, if the anti-inflammatory pathway is confirmed, more targeted treatment could be developed. As this is a small number clinical trial (less than 30 samples), a crossover method was carried out to increase the number according to the features of PBC. Indeed, more validation experiments with increasing number of samples still need to be carried out. In addition, on the aspect of allocation method, although it is blind to patients recruited to the trial with time, our physicians responsible for the trial knew the order before recruiting the second case. In the future study, a random method is also needed.

## Conclusion

TN combined with UDCA improved PBC via inhibition of IL-6 and stimulation of vagal pathway. This suggests the effect of TN combined with UDCA on PBC may be through the cholinergic anti-inflammatory pathway.

## Data Availability

The datasets of the current study are available and can be provided upon reasonable requests from the corresponding author-Zhijun Duan (cathydoctor@sina.com).

## Abbreviations

TN: Transcutaneous Neuromodulation
PBC: Primary Biliary Cirrhosis
HV: Healthy Volunteers
UDCA: Ursodeoxycholic Acid
Bas: Bile Acids
IL-1β: Interleukin-1β
IL-6: Interleukin-6
IL-10: Interleukin-10
TNF-α: Tumor Necrosis Factor-α
ALP: Alkaline Phosphatase
γ-GT: γ-glutamyltransferase
ALT: Alanine Aminotransferase
AST: Aspartate Aminotransferase
TC: Total Cholesterol
TG: Triglyceride
HRV: Heart Rate Variability
TUDCA: Tauroursodeoxycholic Acid
TCA: Taurocholic Acid
TCDCA: Taurochenodeoxycholic Acid
TDCA: Taurodeoxycholic Acid
CDCA: Chenodeoxycholic Acid
CA: Cholic Acid
LCA: Lithocholic Acid
TLCA: Taurolithic Acid
GCDCA: Glyco-eodeoxycholic Acid
GCA: Glycocholic Acid
GDCA: Glycodeoxycholic Acid
DCA: Deoxycholic Acid
LF: Low-frequency
HF: High-frequency
Th1: T helper type 1 cell
Th2: T helper type 2 cell
Th17: T helper type 17 cell
TGF-β: Transforming Growth Factor-beta
